# *In vitro* cell culture models to study hepatitis B and D virus infection

**DOI:** 10.3389/fmicb.2023.1169770

**Published:** 2023-04-05

**Authors:** Hongbo Guo, Stephan Urban, Wenshi Wang

**Affiliations:** ^1^Department of Pathogen Biology and Immunology; Jiangsu Key Laboratory of Immunity and Metabolism, Xuzhou Medical University, Xuzhou, China; ^2^Jiangsu International Laboratory of Immunity and Metabolism, Xuzhou Medical University, Xuzhou, China; ^3^Department of Infectious Diseases, Molecular Virology, University Hospital Heidelberg, Heidelberg, Germany; ^4^German Centre for Infection Research (DZIF), Partner Site Heidelberg, Heidelberg, Germany

**Keywords:** hepatitis B virus, hepatitis D virus, NTCP, virus full life cycle, Infection model

## Abstract

Chronic infection with the hepatitis B virus (HBV) and hepatitis D virus (HDV) can cause a major global health burden. Current medication regimens can repress viral replication and help to control disease progression, but a complete cure is hardly achieved due to the difficulties to eradicate viral templates (cccDNA and integrates). To develop novel curative antiviral therapies for HBV/HDV infection, it is vital to precisely understand the details of the molecular biology of both viruses and the virus-host interactions. One important prerequisite for gaining this aim is the availability of suitable *in vitro* models that support HBV/HDV infection, replicate both viruses *via* their authentic template and allow to adequately study host cell responses. The discovery of sodium taurocholate cotransporting polypeptide (NTCP) receptor as the most crucial host factor promoted HBV/HDV research to a new era. Recently, the structure of human NTCP was solved, gaining a deeper understanding of HBV recognition as the *bona fide* receptor. After decades of continuous efforts, new progress has been achieved in the development of cell culture models supporting HBV/HDV study. This review summarizes the cell culture models currently available, discusses the advantages and disadvantages of each model, and highlights their future applications in HBV and HDV research.

## 1. Introduction

Hepatitis B virus (HBV) is a small, enveloped DNA virus with a high liver tropism and species-specificity. The virus infects hepatocytes through the specific binding to its entry receptor sodium taurocholate cotransporting polypeptide (NTCP; Yan et al., 2012; Ni et al., 2014). After entry into hepatocytes, the nucleocapsid is transported to the nucleus through a possible endocytosis and microtubule-mediated manner. In the nucleus, the relaxed circular DNA (rcDNA) is released and converted into the covalently closed circular DNA (cccDNA). This cccDNA persists as a stable minichromosome and produces all forms of viral transcripts needed for viral protein expression and viral replication (Nkongolo et al., 2019). The 3.5 kb pregenomic RNA (pgRNA) is encapsidated and reverse transcribed into new rcDNA. The newly formed rcDNA-containing capsids are either enveloped and secreted as progeny virions or recycled back to the nucleus for cccDNA pool replenishment (Ko et al., 2018).

In the human liver, HBV-infected hepatocytes can be co-infected with hepatitis D virus (HDV). HDV is a satellite virus of HBV as it requires the HBV envelope proteins to form virus particles. Consequently, in line with HBV, HDV uses the same entry receptor (NTCP) and exhibits a specific liver tropism (Lempp et al., 2017). Of note, HDV can initiate viral replication in cell types other than hepatocytes and even in nonhuman cells, whereas HBV infection can only be established in human hepatocytes due to the requirement of some NTCP-independent human hepatocyte restriction factor(s) (Lempp et al., 2016). Therefore, cell models that are permissive to HBV infection will also support HDV entry and replication (Taylor, 2020). However, the assembly and release of infectious HDV usually need HBV envelope proteins (hepatitis B surface antigen, HBsAg).

Despite an efficient vaccine becomes available since 1980s (WHO, 2017), chronic infection by HBV remains a major public health threat worldwide. Globally, two billion people have been infected with HBV, including 250–300 million chronic carriers (Schweitzer et al., 2015). Up to 30% of chronic HBV patients will develop liver cirrhosis or hepatocellular carcinoma (HCC). Currently, two classes of licensed treatments, reverse transcriptase inhibitors and interferon-α (IFN-α), can effectively control viral replication and prevent disease progression. However, these regimens have little effect on HBV cccDNA. Thus, virus eradication remains rare in chronic HBV patients. HBV/HDV co-infection has been considered as the most severe form of viral hepatitis in humans. Compared to mono-infection of HBV, HBV/HDV co-infection is associated with an accelerated course of liver fibrosis, hepatic decompensation of cirrhosis, and an increased risk for the development of HCC (Wedemeyer, 2010). Averagely, 13% of chronic HBV patients are chronically infected with HDV worldwide (Miao et al., 2020). However, no efficient curative therapies for HDV infection are available.

The availability of reliable cell culture models for the study of HBV and HDV infection is therefore vital to gain insights into the molecular biology of the viruses and, consequently, the development of improved therapeutic medications that resiliently control disease progression or cure HBV and HDV infection. Here we present an overview of the current cell culture models available for the study of HBV and HDV infections.

## 2. Primary hepatocytes

**Figure 1:**
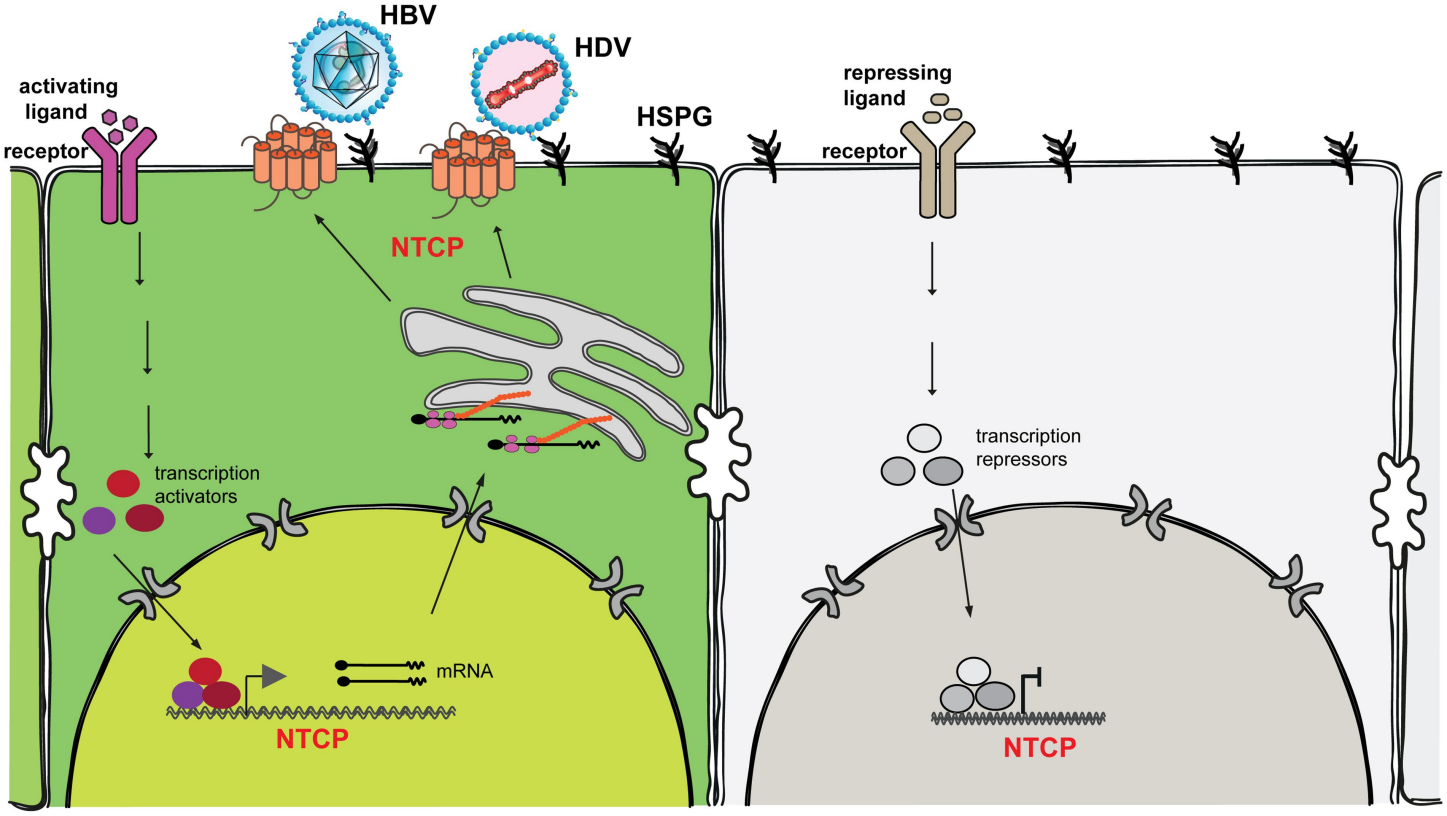
Sodium taurocholate cotransporting polypeptide (NTCP) is a hydrophobic transmembrane glycoprotein containing nine well-ordered transmembrane α-helices with N-terminus at the extracellular surface and C-terminus at the intracellular side (Asami et al., 2022; Liu et al., 2022; Park et al., 2022; Qi and Li, 2022). NTCP is distributed mainly on the basolateral membrane of hepatocytes and functions as an uptake transporter for bile salts. NTCP also essentially supports the entry of HBV and HDV into hepatocytes through the interaction with the preS1 region of the viral envelope protein. Its function is strictly regulated at both the transcriptional and posttranslational levels. Transcriptional Regulation: Substrate (e.g., bile acids), cytokines (e.g., TNF-α, IL-1β, IL-6), liver injury (e.g., cholestasis), and hormones (e.g., prolactin, growth hormone, glucocorticoids, estrogen, thyroid hormone) all regulate transcription of the NTCP gene (Cheng et al., 2007; Bouezzedine et al., 2015; Yan et al., 2019). These ligands bind to their corresponding cellular receptor to kick off signaling transduction, leading to the activation and formation of multiple transcription activators/suppressors to activate/suppress NTCP gene expression. Posttranslational regulation: To become a functional bile salt transporter and an entry receptor for hepatitis B and D viruses, NTCP is destined to be localized to the hepatocyte plasma membrane. This localization is regulated by several posttranslational modification events (e.g., phosphorylation, glycosylation, S-nitrosylation and ubiquitination; Anwer et al., 2005; Kuhlkamp et al., 2005; Schonhoff et al., 2011; Sargiacomo et al., 2018; Zakrzewicz et al., 2022).

For a long time, primary human hepatocytes (PHHs) served as the gold standard *in vitro* model for the study of HBV/HDV infection. In addition, primary hepatocytes isolated from Tupaia belangeri (tree shrews) are also susceptible to HBV and HDV infection (Glebe et al., 2005). Based on the primary Tupaia hepatocytes (PTHs) and a synthetic peptide corresponding to the myristoylated N-terminus of the PreS1 HBV envelope protein, Li and colleagues identified the sodium taurocholate cotransporting polypeptide (NTCP), a transmembrane transporter exclusively localized to the basolateral membrane of the highly differentiated primary hepatocytes, as the *bona fide* receptor for HBV and HDV (Yan et al., 2012). PHHs present the specific metabolism and functionality of the human liver, such as hepatocyte polarization, comprehensive presence of hepatic host factors and preservation of a fully functional innate immune system. Therefore, PHHs are widely used to study HBV/HDV-related host factors, the mode-of-action of antiviral compounds, and the host antiviral responses (Ni et al., 2014; Nkongolo et al., 2014; Watashi et al., 2014; Sato et al., 2015; Verrier et al., 2016; Zhang et al., 2018). Nevertheless, PHHs have a series of limitations that constrain its application of modeling HBV/HDV infection: (1) Limited availability; (2) Despite their regenerative potential in the human body, isolated PHHs do not expand in culture, and are difficult to maintain; (3) PHHs rapidly lose the differentiation status and the susceptibility to HBV/HDV infection shortly after plating (Fig. 1); (4) Due to the divergent host genetic background and the varied quality of the isolated cells, PHHs are highly variable regarding their susceptibility to HBV/HDV infection. Given that PHHs are still the most physiologically relevant *in vitro* model, novel systems or techniques have been developed to improve culture condition and delay the de-differentiation process of the plated PHHs, e.g., collagen sandwich culture (Thomas and Liang, 2016), 3D microfluidic culture (Ortega-Prieto et al., 2018), self-assembling and micro-patterned co-culture with non-parenchymal live cells (Winer et al., 2017), and a five chemicals culture system (Xiang et al., 2019). These improved systems can functionally maintain PHHs to support long-term HBV/HDV infection *in vitro*. However, the exact mechanism related to the differentiation and de-differentiation process of PHHs remains elusive.

## 3. HepaRG cell line

HepaRG, initially derived from a liver tumor with chronic hepatitis C virus infection, was the first hepatoma cell line susceptible to HBV and HDV infection (Gripon et al., 2002). They are bipotent hepatic progenitor cells that can differentiate into biliary-like and hepatocyte-like epithelial cells under a 4-week differentiation protocol. Differentiated HepaRG (dHepaRG) cells have many similarities to PHHs, including polarization, formation of bile canaliculi (Gripon et al., 2002), expression of hepatocyte markers (e.g., HBV/HDV entry receptor, NTCP; Ni et al., 2014), preservation of functional hepatocyte-intrinsic innate immune responses (Luangsay et al., 2015). Therefore, dHepaRG cells have been widely used as a surrogate model to elucidate the viral entry process (Jaoude and Sureau, 2005; Sureau and Salisse, 2013), evaluate antiviral drugs, dissect the mechanism of HBV cccDNA regulation, and analyze innate immune responses during HBV/HDV infection (Mutz et al., 2018; Zhang et al., 2018). Nevertheless, HepaRG cells remain limitations, e.g., a sophisticated long-term differentiation process, supporting limited or no spreading of infection (Hantz et al., 2009), and a relatively low infection efficiency.

## 4. Hepatoma cell lines

HuH7 and HepG2 are hepatoma cell lines that are resistant to HBV and HDV infection due to the loss of NTCP expression (Figure 1). However, the transfection of human hepatoma cell lines with overlength HBV or HDV genome constructs have been used for decades as the first *in vitro* model to study viral replication (Sureau et al., 1986; Kuo et al., 1989). In addition, HepG2 cells were stably transduced with the HBV genome and designated as HepAD38 (Ladner et al., 1997) and HepG2.2.15 (Sells et al., 1987). These two cell models are widely used for the production of HBV inocula, the study of late stage of HBV life cycle (e.g., HBV cccDNA formation and its regulation), and drug screening. For HDV, the co-transfection of hepatoma cells with plasmids encoding HDV genome and HBV envelope proteins fully supports HDV replication and infectious virion secretion (Wang et al., 2021). Given the easy handle of this model, hepatoma cell lines have been widely used to obtain much of today’s knowledge related to HBV and HDV. However, due to their transformed nature, they only partially preserve physiological hepatic function. Therefore, these models are not suitable for studying the full life cycle of HBV and HDV, especially the early steps, including viral entry, internalization and trafficking in hepatocytes.

## 5. Sodium taurocholate cotransporting polypeptide-expressing hepatoma cell lines

Since the discovery of NTCP as the functional entry receptor for HBV and HDV (Yan et al., 2012; Ni et al., 2014), hepatoma cell lines exogenously expressing the human NTCP were created (e.g., HuH7-NTCP, HepG2-NTCP). As easy to access models, these two cell lines enable the systematic identification of new host factors and novel antivirals using the high throughput screening procedures (Urban et al., 2014; Yan et al., 2015; Verrier et al., 2016). In general, HuH7-NTCP cells have a higher susceptibility for HDV mono-infection than HepG2-NTCP cells (which may be attributed to the innate immune incompetence of HuH7 cells). However, for HBV infection, HuH7-NTCP cells are less supportive compared with HepG2-NTCP cells (Ni et al., 2014; Ni and Urban, 2017). HepG2-NTCP cells support the whole life cycle of HBV, from entry to cccDNA formation, establishment of infection, and releasing of progeny virions with low efficiency (Li and Urban, 2016). More profoundly, König et al. (2019) generated a slow proliferating HepG2-NTCP cell clone designated as HepG2-NTCPsec^+^. This clone secrets high levels of infectious HBV progeny virus and supports long-term polyethylene glycol-independent HBV spreading to adjacent cells, resembling key features of HBV spreading *in vivo*.

Currently, despite the supplementation of human NTCP, the establishment of HBV infection in mouse and non-hepatocytic human cell lines is still restricted [with the exception of mouse liver cell line AML12 (Lempp et al., 2016)]. For instance, expression of human NTCP in mouse hepatocytes enables HBV entry, but subsequently cccDNA does not form in most murine cells (Lempp et al., 2016). Notably, Lei and his colleagues reported that the conversion of rcDNA to cccDNA was supported in murine cells. This indicates that the HBV life cycle is blocked post entry and likely before the repair stage in mouse cells (Wei and Ploss, 2020; Wei and Ploss, 2021; Wei et al., 2022). In contrast, the complementation of non-liver cell lines or even non-human hepatocytes with human NTCP confers susceptibility to HDV (Lempp et al., 2016, 2017). Nevertheless, Giersch et al. (2021); Giersch and Dandri (2021) showed that murine hepatocytes with humanized NTCP, although supporting HDV infection, failed to support the persistence of HDV mono-infection *in vivo*, while human hepatocytes maintained HDV infection for at least 42 days. This indicates that besides the entry receptor NTCP, additional species-specific factors are probably needed to maintain HDV persistence.

## 6. Sodium taurocholate cotransporting polypeptide- and HBsAg-expressing hepatoma cell lines

HDV, as a satellite virus of HBV, uses the HBV-encoded envelope proteins (large, middle and small-HBsAg) for progeny virus assembly, release and *de novo* entry into hepatocytes. All human NTCP-expressing cell lines support HDV entry, viral replication and expression of HDAg. However, subsequent steps including assembly and release of progeny virus are blocked due to the lack of HBV envelope proteins (Lempp and Urban, 2017). By stably transducing HepG2 cells with genes encoding NTCP and the HBV envelope proteins, Lempp et al. (2019) produced a cell line (designated as HepNB2.7) that allows continuous secretion of infectious progeny HDV following primary infection, thus supporting the complete life cycle of HDV. In addition, Ni and his colleagues created a novel cell line named HuH7-END through stepwise integration of the cDNA of the HDV antigenome, the genes of HBV envelope proteins and the NTCP-receptor. This enables HuH7-END cells to support continuous replication of HDV, high levels of HDV secretion and *de novo* HDV entry and spreading (Ni et al., 2019). These two novel models are applicable as a screening platform to determine potential antiviral drugs that target any stage of HDV life cycle (especially the later stages, including release and assembly) and HBsAg secretion. Notably, for HuH7-END cells, virus secretion and production is stable for > 16 passages and can be used for large-scale virus production (Ni et al., 2019). Interestingly, although the preS1 region of the L-protein irreversibly binds to NTCP with a high affinity, the stable co-expression of NTCP and the HBV envelope proteins within one cell can still result in two separated and functional proteins. This implies that HBsAg might be protected from interacting with NTCP during protein synthesis and transport or the binding competence of these two proteins is hidden before reaching their final location. This important observation paves the way for the successful generation of HepNB2.7 and HuH7-END cells.

## 7. Stem cell-derived hepatocytes

In addition to the efforts in generating HBV/HDV susceptible cell lines, stem cell-derived hepatocytes also represent a promising model for HBV/HDV research (Ni and Urban, 2017). Hepatocyte-like cells (HLCs) are differentiated *in vitro* from diverse resources, such as human embryonic stem cells (hESMs), induced pluripotent human stem cells (iPSCs), liver-resident hepatic progenitor cells, and bone marrow-derived mesenchymal stem cells (Wang et al., 2019). After differentiation, these cells endogenously express hepatic markers, like retinoic X receptor (RXR), hepatocyte nuclear factor 4 alpha (HNF4α) and the NTCP receptor, thus being susceptible to HBV infection (Shlomai et al., 2014; Kaneko et al., 2016). Importantly, based on an optimized protocol, Xia and his colleagues reported that HLCs can maintain the susceptible state for up to 4 weeks after differentiation, making HLCs superior compared with PHHs. Also, this slowed dedifferentiation process becomes a prerequisite for HLCs to model long-term HBV infection and support a low level of virus spreading (Xia et al., 2017). Culture conditions in 2D systems fail to mimic the complexity and architecture of the liver microenvironment, thus prohibiting the interrogation of functional roles of the extracellular matrix, and/or the impact of different cell types on virus infection/spreading (Gural et al., 2018). In light of these limitations, Nie et al. (2018) used iPSCs to generate a functional 3D liver organoid (LO). Compared with 2D cultured HLCs, the organoids exhibit stronger hepatic functions, possess more susceptibility to HBV infection, maintain HBV propagation and produce infectious viruses for a prolonged duration. Notably, hiPSC-derived LOs could also inherit the genetic background of the donor, and recapitulate virus-induced hepatic dysfunction (e.g., down-regulation of hepatic gene expression, release of early acute liver failure markers, and altered hepatic ultrastructure). These observations suggest that LOs may serve as a potential personalized model for the study of HBV/HDV infection and virus-host interaction.

## 8. Conclusion

Although HBV and HDV have been identified for decades (Blumberg et al., 1965; Rizzetto et al., 1977), many fundamental questions remain poorly understood. For instance, the molecular mechanism of virus entry, the hepatocyte-specific factors involved in the infection process and cccDNA regulation, virus release and spreading, innate immune sensing, and signaling pathways involved in HBV/HDV-induced HCC. Nevertheless, continuous progress has been made in the development of *in vitro* models to support HBV/HDV studies. The identification of the HBV/HDV entry receptor NTCP was a significant milestone leading to the generation of HBV/HDV permissive hepatoma cell lines. These NTCP-expressing cells (e.g., HepG2-NTCP) support the major infection steps, from HBV/HDV entry to HBV cccDNA formation, HBV/HDV replication, HBV/HDV protein expression and HBV virion assembly. Therefore, these models are amendable for high throughput screening procedures to systematically identify new factors involved in HBV/HDV infection and the development of new antivirals. More profoundly, the human stem cell-derived hepatocyte (Xia et al., 2017) and a HepG2-NTCP derived cell clone (HepG2-NTCPsec +; König et al., 2019) could also support HBV spreading, which occurs efficiently *in vivo*. For HDV, all human NTCP-expressing cells do not support its complete life cycle due to the lack of HBsAg. Recently, NTCP- and HBsAg-expressing hepatoma cell lines HepNB2.7 (Lempp et al., 2019; Wang et al., 2021) and HuH7-END (Ni et al., 2019), which support the full life cycle of HDV, have been successfully established. These state-of-art cell culture models together with the conventional systems offer both academic centers and pharmaceutical industries good opportunities to choose suitable models on the basis of specific questions addressed (Table 1).

**Table 1:** Cell culture models for HBV and HDV infection.

Model	Entry	Replication	Secretion	*de novo* spread	Comments
PHH	HBV(+++) HDV(++)	HBV(+++) HDV(+++)	HBV(+)^1^ HDV(+)^1^^,^^2^	HBV(+)^1^ HDV(+)^1^^,^^2^	Pros: Gold standard in HBV/HDV infection; closest resemblance of hepatic metabolism and innate immune signaling Cons: Limited availability; high donor-to-doner variability
HepaRG	HBV(+) HDV(+)	HBV(+++) HDV(+++)	HBV(+) HDV(+)^2^	HBV(−) HDV(−)	Pros: Physiologically close to PHH; competent innate immune signaling Cons: Requires sophisticated differentiation protocol; differentiates into biliary and hepatic cells; relatively low infection rate
HuH7 (transfection)	HBV(−) HDV(−)	HBV(++) HDV(+++)	HBV(++) HDV(+++)^3^	HBV(−) HDV(−)	Pros: easy handling; widely available Cons: does not reflect authentic infection, especially the early stage of viral entry, internalization and trafficking in hepatocytes
HepAD38/HepG2.2.15	HBV(−)	HBV(+++)	HBV(+++)	HBV(−)	Pros: easy handling; suitable for the production of HBV inoculate stock; feasible for drug screening that targets the late stage of HBV life cycle Cons: only partially resemble hepatocytes; does not reflect authentic HBV infection
HepG2-NTCP	HBV(+++) HDV(++)	HBV(+++) HDV(++)	HBV(+) HDV(++)^2^	HBV(−) HDV(−)	Pros: easy handling; efficient infection rate for HBV and HDV; suitable for high throughput screening Cons: only partially resemble hepatocytes
HuH7-NTCP	HBV(+++) HDV(++)	HBV(++) HDV(+++)	HBV(+) HDV(++)^2^	HBV(−) HDV(−)	Pros: easy handling; efficient infection rate for HDV; suitable for HDV-related high throughput screening Cons: only partially resemble hepatocytes; low infection rate for HBV; deficient in some aspects of innate immune signaling
HepG2-NTCPsec^+^	HBV(+++) HDV(++)	HBV(+++) HDV(++)	HBV(++) HDV(++)^2^	HBV(++) HDV(++)^2^	Pros: supports the complete HBV life cycle, and long-term viral spread; supports the propagation of patient-derived HBV Cons: only partially resemble hepatocytes
HepNB2.7	HDV(++)	HDV(+++)	HDV(+++)	HDV(+)	Pros: supports the complete HDV replication cycle, suitable for antiviral drug screening, especially for drugs interfering with late steps of the HDV life cycle, e.g., virion assembly and secretion; can be used to identify compounds that affect HBsAg secretion Cons: only partially resemble hepatocytes
HuH7-END	HDV(++)	HDV(+++)	HDV(+++)	HDV(+)	Pros: can be easily scaled up for preparation of large HDV virus stocks; suitable for screening of antiviral drugs targeting HDV replication; can be used to identify compounds that affect HBsAg secretion Cons: only partially resemble hepatocytes
Stem cell-derived hepatocytes	HBV(++) HDV(++)	HBV(++) HDV(++)	HBV(++) HDV(++)^2^	HBV(+) HDV(+)^2^	Pros: support long-term infection; physiologically close to PHH; support limited virus spread; with a better performance in 3D culture; serves as a potential personalized model Cons: requires elaborate differentiation protocol

1 Long-term functional maintenance of PHH;

2 when co-infected with HBV; and

3 when co-transfected with a plasmid containing HBV genome or encoding HBsAg.

(−) no, (+) yes, low, (++) yes, moderate, (+++) yes, high.

Ideally, the most physiological *in vitro* cell model is to endogenously express hepatic markers, e.g., retinoic X receptor (RXR), hepatocyte nuclear factor 4 alpha (HNF4α) and the NTCP receptor, thus maintaining the hepatic properties. However, hepatocytes rapidly lose the differentiation status once cultured *in vitro*, representing the main obstacle to developing the optimal cell culture models. Regrettably, the exact mechanism related to the differentiation and de-differentiation process of hepatocytes remains largely elusive. Herein, we summarized the possible ways to regulate the expression of endogenous NTCP at both transcriptional and posttranslational levels (Figure 1). If all the key cellular elements or pathways involved in the expression of main hepatic markers are elucidated, this would significantly revolutionize the development of cell culture models for HBV and HDV. Ultimately, this significant progress will contribute to the understanding of the key aspects of HBV/HDV and pave the way for the identification of new antivirals that target the different steps of HBV/HDV life cycle in the future (Urban et al., 2021; Dusheiko et al., 2023).

## Author contributions

HG, SU, and WW wrote the manuscript. All authors contributed to the article and approved the submitted version.

## Funding

This work was funded by the National Natural Science Foundation of China (32270161, 32100117, 32100118), the Natural Science Foundation of Jiangsu Province of China (BK20210899, BK20210900), Research Grant of Jiangsu Commission of Health, China (ZD2021036), and the Starting Grant for Talents of Xuzhou Medical University (D2021007, D2021008).

## Conflict of interest

SU is co-applicant and co-inventor of patents protecting Myrcludex B.

The remaining authors declare that the research was conducted in the absence of any commercial or financial relationships that could be construed as a potential conflict of interest.

## Publisher’s note

All claims expressed in this article are solely those of the authors and do not necessarily represent those of their affiliated organizations, or those of the publisher, the editors and the reviewers. Any product that may be evaluated in this article, or claim that may be made by its manufacturer, is not guaranteed or endorsed by the publisher.

## References

[ref1] Anwer M. S. Gillin H. Mukhopadhyay S. Balasubramaniyan N. Suchy F. J. Ananthanarayanan M. (2005). Dephosphorylation of Ser-226 facilitates plasma membrane retention of Ntcp. J. Biol. Chem. 280, 33687–33692. doi: 10.1074/jbc.M502151200, PMID: 16027164

[ref2] Asami J. Kimura K. T. Fujita-Fujiharu Y. Ishida H. Zhang Z. Nomura Y.. (2022). Structure of the bile acid transporter and HBV receptor NTCP. Nature 606, 1021–1026. doi: 10.1038/s41586-022-04845-4, PMID: 35580629

[ref3] Blumberg B. S. Alter H. J. Visnich S. (1965). A "New" Antigen in Leukemia Sera. JAMA 191, 541–546. doi: 10.1001/jama.1965.03080070025007 14239025

[ref4] Bouezzedine F. Fardel O. Gripon P. (2015). Interleukin 6 inhibits HBV entry through NTCP down regulation. Virology 481, 34–42. doi: 10.1016/j.virol.2015.02.026, PMID: 25765005

[ref5] Cheng X. Buckley D. Klaassen C. D. (2007). Regulation of hepatic bile acid transporters Ntcp and Bsep expression. Biochem. Pharmacol. 74, 1665–1676. doi: 10.1016/j.bcp.2007.08.014, PMID: 17897632 PMC2740811

[ref6] Dusheiko G. Agarwal K. Maini M. K. (2023). New approaches to chronic hepatitis B. N. Engl. J. Med. 388, 55–69. doi: 10.1056/NEJMra2211764, PMID: 36599063

[ref7] Giersch K. Dandri M. (2021). In vivo models of HDV infection: is humanizing NTCP enough? Viruses 13, 410–419. doi: 10.3390/v13040588, PMID: 33807170 PMC8065588

[ref8] Giersch K. Hermanussen L. Volz T. Kah J. Allweiss L. Casey J.. (2021). Murine hepatocytes do not support persistence of hepatitis D virus mono-infection in vivo. Liver Int. 41, 410–419. doi: 10.1111/liv.14677 32997847

[ref9] Glebe D. Urban S. Knoop E. V. Çagˇ N. Krass P. Grün S.. (2005). Mapping of the hepatitis B virus attachment site by use of infection-inhibiting preS1 lipopeptides and tupaia hepatocytes. Gastroenterology 129, 234–245. doi: 10.1053/j.gastro.2005.03.090, PMID: 16012950

[ref10] Gripon P. Rumin S. Urban S. le Seyec J. Glaise D. Cannie I.. (2002). Infection of a human hepatoma cell line by hepatitis B virus. Proc. Natl. Acad. Sci. U. S. A. 99, 15655–15660. doi: 10.1073/pnas.232137699, PMID: 12432097 PMC137772

[ref11] Gural N. Mancio-Silva L. He J. Bhatia S. N. (2018). Engineered livers for infectious diseases. Cell. Mol. Gastroenterol. Hepatol. 5, 131–144. doi: 10.1016/j.jcmgh.2017.11.005, PMID: 29322086 PMC5756057

[ref12] Hantz O. Parent R. Durantel D. Gripon P. Guguen-Guillouzo C. Zoulim F. (2009). Persistence of the hepatitis B virus covalently closed circular DNA in HepaRG human hepatocyte-like cells. J. Gen. Virol. 90, 127–135. doi: 10.1099/vir.0.004861-0, PMID: 19088281

[ref13] Jaoude G. A. Sureau C. (2005). Role of the antigenic loop of the hepatitis B virus envelope proteins in infectivity of hepatitis delta virus. J. Virol. 79, 10460–10466. doi: 10.1128/JVI.79.16.10460-10466.2005, PMID: 16051838 PMC1182656

[ref14] Kaneko S. Kakinuma S. Asahina Y. Kamiya A. Miyoshi M. Tsunoda T.. (2016). Human induced pluripotent stem cell-derived hepatic cell lines as a new model for host interaction with hepatitis B virus. Sci. Rep. 6:29358. doi: 10.1038/srep29358, PMID: 27386799 PMC4937433

[ref15] Ko C. Chakraborty A. Chou W. M. Hasreiter J. Wettengel J. M. Stadler D.. (2018). Hepatitis B virus genome recycling and de novo secondary infection events maintain stable cccDNA levels. J. Hepatol. 69, 1231–1241. doi: 10.1016/j.jhep.2018.08.012, PMID: 30142426 PMC7611400

[ref16] König A. Yang J. Jo E. Park K. H. P. Kim H. Than T. T.. (2019). Efficient long-term amplification of hepatitis B virus isolates after infection of slow proliferating HepG2-NTCP cells. J. Hepatol. 71, 289–300. doi: 10.1016/j.jhep.2019.04.010, PMID: 31077792

[ref17] Kuhlkamp T. Keitel V. Helmer A. Haussinger D. Kubitz R. (2005). Degradation of the sodium taurocholate cotransporting polypeptide (NTCP) by the ubiquitin-proteasome system. Biol. Chem. 386, 1065–1074. doi: 10.1515/BC.2005.122, PMID: 16218878

[ref18] Kuo M. Y. Chao M. Taylor J. (1989). Initiation of replication of the human hepatitis delta virus genome from cloned DNA: role of delta antigen. J. Virol. 63, 1945–1950. doi: 10.1128/jvi.63.5.1945-1950.1989, PMID: 2649689 PMC250607

[ref19] Ladner S. K. Otto M. J. Barker C. S. Zaifert K. Wang G. H. Guo J. T.. (1997). Inducible expression of human hepatitis B virus (HBV) in stably transfected hepatoblastoma cells: a novel system for screening potential inhibitors of HBV replication. Antimicrob. Agents Chemother. 41, 1715–1720. doi: 10.1128/AAC.41.8.1715, PMID: 9257747 PMC163991

[ref20] Lempp F. A. Mutz P. Lipps C. Wirth D. Bartenschlager R. Urban S. (2016). Evidence that hepatitis B virus replication in mouse cells is limited by the lack of a host cell dependency factor. J. Hepatol. 64, 556–564. doi: 10.1016/j.jhep.2015.10.030, PMID: 26576481

[ref21] Lempp F. A. Qu B. Wang Y. X. Urban S. (2016). Hepatitis B virus infection of a mouse hepatic cell line reconstituted with human sodium taurocholate cotransporting polypeptide. J. Virol. 90, 4827–4831. doi: 10.1128/JVI.02832-15, PMID: 26865711 PMC4836309

[ref22] Lempp F. A. Schlund F. Rieble L. Nussbaum L. Link C. Zhang Z.. (2019). Recapitulation of HDV infection in a fully permissive hepatoma cell line allows efficient drug evaluation. Nat. Commun. 10:2265. doi: 10.1038/s41467-019-10211-2, PMID: 31118422 PMC6531471

[ref23] Lempp F. A. Urban S. (2017). Hepatitis Delta virus: replication strategy and upcoming therapeutic options for a neglected human pathogen. Viruses 9:172. doi: 10.3390/v9070172, PMID: 28677645 PMC5537664

[ref24] Lempp F. A. Wiedtke E. Qu B. Roques P. Chemin I. Vondran F. W. R.. (2017). Sodium taurocholate cotransporting polypeptide is the limiting host factor of hepatitis B virus infection in macaque and pig hepatocytes. Hepatology 66, 703–716. doi: 10.1002/hep.29112, PMID: 28195359

[ref25] Li W. Urban S. (2016). Entry of hepatitis B and hepatitis D virus into hepatocytes: basic insights and clinical implications. J. Hepatol. 64, S32–S40. doi: 10.1016/j.jhep.2016.02.011, PMID: 27084034 PMC7114860

[ref26] Liu H. Irobalieva R. N. Bang-Sørensen R. Nosol K. Mukherjee S. Agrawal P.. (2022). Structure of human NTCP reveals the basis of recognition and sodium-driven transport of bile salts into the liver. Cell Res. 32, 773–776. doi: 10.1038/s41422-022-00680-4, PMID: 35726088 PMC9343345

[ref27] Luangsay S. Ait-Goughoulte M. Michelet M. Floriot O. Bonnin M. Gruffaz M.. (2015). Expression and functionality of toll- and RIG-like receptors in HepaRG cells. J. Hepatol. 63, 1077–1085. doi: 10.1016/j.jhep.2015.06.022, PMID: 26144659

[ref28] Miao Z. Zhang S. Ou X. Li S. Ma Z. Wang W.. (2020). Estimating the global prevalence, disease progression, and clinical outcome of Hepatitis Delta virus infection. J. Infect. Dis. 221, 1677–1687. doi: 10.1093/infdis/jiz633, PMID: 31778167 PMC7184909

[ref29] Mutz P. Metz P. Lempp F. A. Bender S. Qu B. Schöneweis K.. (2018). HBV bypasses the innate immune response and does not protect HCV from antiviral activity of interferon. Gastroenterology 154, 1791–1804.e22. doi: 10.1053/j.gastro.2018.01.044, PMID: 29410097

[ref30] Ni Y. Lempp F. A. Mehrle S. Nkongolo S. Kaufman C. Fälth M.. (2014). Hepatitis B and D viruses exploit sodium taurocholate co-transporting polypeptide for species-specific entry into hepatocytes. Gastroenterology 146, 1070–1083.e6. doi: 10.1053/j.gastro.2013.12.024, PMID: 24361467

[ref31] Ni Y. Urban S. (2017). Stem cell-derived hepatocytes: a promising novel tool to study hepatitis B virus infection. J. Hepatol. 66, 473–475. doi: 10.1016/j.jhep.2016.11.027, PMID: 27965155

[ref32] Ni Y. Zhang Z. Engelskircher L. Verch G. Tu T. Lempp F. A.. (2019). Generation and characterization of a stable cell line persistently replicating and secreting the human hepatitis delta virus. Sci. Rep. 9:10021. doi: 10.1038/s41598-019-46493-1, PMID: 31292511 PMC6620269

[ref33] Nie Y. Z. Zheng Y. W. Miyakawa K. Murata S. Zhang R. R. Sekine K.. (2018). Recapitulation of hepatitis B virus-host interactions in liver organoids from human induced pluripotent stem cells. EBioMedicine 35, 114–123. doi: 10.1016/j.ebiom.2018.08.014, PMID: 30120080 PMC6156717

[ref34] Nkongolo S. Lempp F. A. Wodrich H. Urban S. Ni Y. Ni Y. (2019). The retinoic acid receptor (RAR) alpha-specific agonist Am80 (tamibarotene) and other RAR agonists potently inhibit hepatitis B virus transcription from cccDNA. Antivir. Res. 168, 146–155. doi: 10.1016/j.antiviral.2019.04.009, PMID: 31018112

[ref35] Nkongolo S. Ni Y. Lempp F. A. Kaufman C. Lindner T. Esser-Nobis K.. (2014). Cyclosporin a inhibits hepatitis B and hepatitis D virus entry by cyclophilin-independent interference with the NTCP receptor. J. Hepatol. 60, 723–731. doi: 10.1016/j.jhep.2013.11.022, PMID: 24295872

[ref36] Ortega-Prieto A. M. Skelton J. K. Wai S. N. Large E. Lussignol M. Vizcay-Barrena G.. (2018). 3D microfluidic liver cultures as a physiological preclinical tool for hepatitis B virus infection. Nat. Commun. 9:682. doi: 10.1038/s41467-018-02969-8, PMID: 29445209 PMC5813240

[ref37] Park J. H. Iwamoto M. Yun J. H. Uchikubo-Kamo T. Son D. Jin Z.. (2022). Structural insights into the HBV receptor and bile acid transporter NTCP. Nature 606, 1027–1031. doi: 10.1038/s41586-022-04857-0, PMID: 35580630 PMC9242859

[ref38] Qi X. Li W. (2022). Unlocking the secrets to human NTCP structure. Innovation (Camb) 3:100294. doi: 10.1016/j.xinn.2022.100294 36032196 PMC9399531

[ref39] Rizzetto M. Canese M. G. Arico S. Crivelli O. Trepo C. Bonino F.. (1977). Immunofluorescence detection of new antigen-antibody system (delta/anti-delta) associated to hepatitis B virus in liver and in serum of HBsAg carriers. Gut 18, 997–1003. doi: 10.1136/gut.18.12.997, PMID: 75123 PMC1411847

[ref40] Sargiacomo C. el-Kehdy H. Pourcher G. Stieger B. Najimi M. Sokal E. (2018). Age-dependent glycosylation of the sodium taurocholate cotransporter polypeptide: from fetal to adult human livers. Hepatol. Commun. 2, 693–702. doi: 10.1002/hep4.1174, PMID: 29881821 PMC5983131

[ref41] Sato S. Li K. Kameyama T. Hayashi T. Ishida Y. Murakami S.. (2015). The RNA sensor RIG-I dually functions as an innate sensor and direct antiviral factor for hepatitis B virus. Immunity 42, 123–132. doi: 10.1016/j.immuni.2014.12.016, PMID: 25557055

[ref42] Schonhoff C. M. Ramasamy U. Anwer M. S. (2011). Nitric oxide-mediated inhibition of taurocholate uptake involves S-nitrosylation of NTCP. Am. J. Physiol. Gastrointest. Liver Physiol. 300, G364–G370. doi: 10.1152/ajpgi.00170.2010, PMID: 21109590 PMC3043645

[ref43] Schweitzer A. Horn J. Mikolajczyk R. T. Krause G. Ott J. J. (2015). Estimations of worldwide prevalence of chronic hepatitis B virus infection: a systematic review of data published between 1965 and 2013. Lancet 386, 1546–1555. doi: 10.1016/S0140-6736(15)61412-X, PMID: 26231459

[ref44] Sells M. A. Chen M. L. Acs G. (1987). Production of hepatitis B virus particles in Hep G2 cells transfected with cloned hepatitis B virus DNA. Proc. Natl. Acad. Sci. U. S. A. 84, 1005–1009. doi: 10.1073/pnas.84.4.1005, PMID: 3029758 PMC304350

[ref45] Shlomai A. Schwartz R. E. Ramanan V. Bhatta A. de Jong Y. P. Bhatia S. N.. (2014). Modeling host interactions with hepatitis B virus using primary and induced pluripotent stem cell-derived hepatocellular systems. Proc. Natl. Acad. Sci. U. S. A. 111, 12193–12198. doi: 10.1073/pnas.1412631111, PMID: 25092305 PMC4143014

[ref46] Sureau C. Romet-Lemonne J. L. Mullins J. I. Essex M. (1986). Production of hepatitis B virus by a differentiated human hepatoma cell line after transfection with cloned circular HBV DNA. Cells 47, 37–47. doi: 10.1016/0092-8674(86)90364-8, PMID: 3019565

[ref47] Sureau C. Salisse J. (2013). A conformational heparan sulfate binding site essential to infectivity overlaps with the conserved hepatitis B virus a-determinant. Hepatology 57, 985–994. doi: 10.1002/hep.26125, PMID: 23161433

[ref48] Taylor J. M. (2020). Infection by Hepatitis Delta virus. Viruses 12:648. doi: 10.3390/v12060648 32560053 PMC7354607

[ref49] Thomas E. Liang T. J. (2016). Experimental models of hepatitis B and C–new insights and progress. Nat. Rev. Gastroenterol. Hepatol. 13, 362–374. doi: 10.1038/nrgastro.2016.37, PMID: 27075261 PMC5578419

[ref50] Urban S. Bartenschlager R. Kubitz R. Zoulim F. (2014). Strategies to inhibit entry of HBV and HDV into hepatocytes. Gastroenterology 147, 48–64. doi: 10.1053/j.gastro.2014.04.030, PMID: 24768844

[ref51] Urban S. Neumann-Haefelin C. Lampertico P. (2021). Hepatitis D virus in 2021: virology, immunology and new treatment approaches for a difficult-to-treat disease. Gut 70, 1782–1794. doi: 10.1136/gutjnl-2020-323888, PMID: 34103404 PMC8355886

[ref52] Verrier E. R. Colpitts C. C. Bach C. Heydmann L. Weiss A. Renaud M.. (2016). A targeted functional RNA interference screen uncovers glypican 5 as an entry factor for hepatitis B and D viruses. Hepatology 63, 35–48. doi: 10.1002/hep.28013, PMID: 26224662

[ref53] Wang W. Lempp F. A. Schlund F. Walter L. Decker C. C. Zhang Z.. (2021). Assembly and infection efficacy of hepatitis B virus surface protein exchanges in 8 hepatitis D virus genotype isolates. J. Hepatol. 75, 311–323. doi: 10.1016/j.jhep.2021.03.025, PMID: 33845061

[ref54] Wang J. Qu B. Zhang F. Zhang C. Deng W. Dao Thi V. L.. (2019). Stem cell-derived hepatocyte-like cells as model for viral hepatitis research. Stem Cells Int. 2019, 1–11. doi: 10.1155/2019/9605252 PMC6594266 31281392

[ref55] Watashi K. Sluder A. Daito T. Matsunaga S. Ryo A. Nagamori S.. (2014). Cyclosporin a and its analogs inhibit hepatitis B virus entry into cultured hepatocytes through targeting a membrane transporter, sodium taurocholate cotransporting polypeptide (NTCP). Hepatology 59, 1726–1737. doi: 10.1002/hep.26982, PMID: 24375637 PMC4265264

[ref56] Wedemeyer H. (2010). Re-emerging interest in hepatitis delta: new insights into the dynamic interplay between HBV and HDV. J. Hepatol. 52, 627–629. doi: 10.1016/j.jhep.2010.02.001, PMID: 20334947

[ref57] Wei L. Cafiero T. R. Tseng A. Gertje H. P. Berneshawi A. Crossland N. A.. (2022). Conversion of hepatitis B virus relaxed circular to covalently closed circular DNA is supported in murine cells. JHEP Rep. 4:100534. doi: 10.1016/j.jhepr.2022.100534, PMID: 36035363 PMC9403495

[ref58] Wei L. Ploss A. (2020). Core components of DNA lagging strand synthesis machinery are essential for hepatitis B virus cccDNA formation. Nat. Microbiol. 5, 715–726. doi: 10.1038/s41564-020-0678-0, PMID: 32152586 PMC7190442

[ref59] Wei L. Ploss A. (2021). Mechanism of hepatitis B virus cccDNA formation. Viruses 13:1463. doi: 10.3390/v13081463, PMID: 34452329 PMC8402782

[ref60] WHO (2017). Hepatitis B vaccines: WHO position paper–July 2017. Wkly Epidemiol. Rec. 92, 369–392. 28685564

[ref61] Winer B. Y. Huang T. S. Pludwinski E. Heller B. Wojcik F. Lipkowitz G. E.. (2017). Long-term hepatitis B infection in a scalable hepatic co-culture system. Nat. Commun. 8:125. doi: 10.1038/s41467-017-00200-8, PMID: 28743900 PMC5527081

[ref62] Xia Y. Carpentier A. Cheng X. Block P. D. Zhao Y. Zhang Z.. (2017). Human stem cell-derived hepatocytes as a model for hepatitis B virus infection, spreading and virus-host interactions. J. Hepatol. 66, 494–503. doi: 10.1016/j.jhep.2016.10.009, PMID: 27746336 PMC5316493

[ref63] Xiang C. du Y. Meng G. Soon Yi L. Sun S. Song N.. (2019). Long-term functional maintenance of primary human hepatocytes in vitro. Science 364, 399–402. doi: 10.1126/science.aau7307, PMID: 31023926

[ref64] Yan Y. Allweiss L. Yang D. Kang J. Wang J. Qian X.. (2019). Down-regulation of cell membrane localized NTCP expression in proliferating hepatocytes prevents hepatitis B virus infection. Emerg. Microbes Infect. 8, 879–894. doi: 10.1080/22221751.2019.1625728, PMID: 31179847 PMC6567113

[ref65] Yan H. Liu Y. Sui J. Li W. (2015). NTCP opens the door for hepatitis B virus infection. Antivir. Res. 121, 24–30. doi: 10.1016/j.antiviral.2015.06.002, PMID: 26071008

[ref66] Yan H. Zhong G. Xu G. He W. Jing Z. Gao Z.. (2012). Sodium taurocholate cotransporting polypeptide is a functional receptor for human hepatitis B and D virus. elife 1:e00049. doi: 10.7554/eLife.00049, PMID: 23150796 PMC3485615

[ref67] Zakrzewicz D. Leidolf R. Kunz S. Müller S. F. Neubauer A. Leiting S.. (2022). Tyrosine 146 of the human Na(+)/taurocholate Cotransporting polypeptide (NTCP) is essential for its hepatitis B virus (HBV) receptor function and HBV entry into hepatocytes. Viruses 14:1259. doi: 10.3390/v14061259, PMID: 35746730 PMC9230856

[ref68] Zhang Z. Filzmayer C. Ni Y. Sültmann H. Mutz P. Hiet M. S.. (2018). Hepatitis D virus replication is sensed by MDA5 and induces IFN-beta/lambda responses in hepatocytes. J. Hepatol. 69, 25–35. doi: 10.1016/j.jhep.2018.02.021, PMID: 29524530

